# Association of serum uric acid with prognosis in patients with myocardial infarction: an update systematic review and meta-analysis

**DOI:** 10.1186/s12872-023-03523-1

**Published:** 2023-10-17

**Authors:** Jiacheng Rong, Cheng Fang, Xudong Chen, Chaokun Hong, Lei Huang

**Affiliations:** 1 Cardiovascular Department, Ningbo Hangzhou Bay Hospital, Qianwan New District, Ningbo, Zhejiang China; 2 Department of Urologic Surgery, Ningbo Urology and Nephrology Hospital, Ningbo Yinzhou No.2 Hospital, Ningbo, Zhejiang China; 3 MRC Centre for Global Infectious Disease Analysis Faculty of Medicine, Imperial College, London, UK

**Keywords:** Myocardial infarction, Serum uric acid, Hyperuricemia, Prognosis, Meta-analysis, Systematic review

## Abstract

**Background:**

The prognostic significance of serum uric acid (SUA) in individuals who have experienced myocardial infarction (MI) remains a subject of academic debate. Thus, the aim of this study was to examine the occurrence of immediate and long-term adverse outcomes in individuals with elevated levels of uric acid (UA) following a diagnosis of MI.

**Method:**

This study conducted a literature search from PubMed, Embase, Web of Science, Medline, Cochrane Library, Emcrae, and Scopus to perform a systematic review and meta-analysis of the prognostic impact of MI with a hyper SUA to assess short-term (30-day or in-hospital) and long-term mortality, the incidence of major adverse cardiovascular events (MACE), and its adverse event rate in relation to SUA. The literature search was conducted up until April 2023. A random effects model and risk ratio (RR) were used as epidemiological indicators. For indicators with low disease rates, treatment intensity was reduced and RR was considered equivalent to odds ratio (OR). Hazard Ratio (HR), RR, and OR extracted from the data were simultaneously subjected to multivariable adjustment for confounding factors. In addition, *P* values for all original hypotheses were extracted and a meta-analysis was conducted. High SUA was defined as SUA levels equal to or greater than 420 μmol/L (7.0 mg/dL) for males and equal to or greater than 357 μmol/L (6.0 mg/dL) for females. The quality of the literature was evaluated using the Newcastle–Ottawa Scale (NOS).

**Results:**

This comprehensive study included a total of 41 investigations, involving a large sample size of 225,600 individuals who had experienced MI. The findings from the meta-analysis reveal that patients diagnosed with hyperuricemia have significantly increased rates of short-term mortality (RR = 2.14, 95% CI = 1.86, 2.48) and short-term incidence of MACE (RR = 1.94, 95% CI = 1.65–2.11). Furthermore, long-term adverse outcomes, including all-cause mortality (RR = 1.46, 95% CI = 1.40–1.51) and incidence of MACE (RR = 1.43, 95% CI = 1.35–1.52), were also found to be higher in this specific patient population.

**Conclusion:**

Patients diagnosed with MI and elevated SUA levels exhibit a heightened incidence of MACE during their hospital stay. Furthermore, these individuals also experience elevated rates of in-hospital mortality and mortality within one year of hospitalization. However, it is important to note that further randomized controlled trials are necessary to validate and authenticate these findings.

**Supplementary Information:**

The online version contains supplementary material available at 10.1186/s12872-023-03523-1.

## Introduction

UA serves as the end product of purine metabolism in thehuman body. Its production is facilitated by the enzyme xanthine oxidase (XO), which not only plays a role in UA synthesis but also acts as a source of reactive oxygen species (ROS) in the cardiovascular system [1, 2]. Xanthine oxidase is an enzyme widely distributed throughout different human body organs [3]. Additionally, the enzyme uricase has undergone genetic mutations in humans, resulting in circulating UA levels 3 to 10 times higher than in other mammals [4].

A correlation has been observed between heightened levels of SUA and various pathological processes, including but not limited to heightened oxidative stress, inflammation, and endothelial dysfunction [5]. Consequently, elevated SUA levels are frequently linked to unfavourable outcomes in diverse cardiovascular conditions [6]. UA undergoes a swift escalation within the tissues, subsequently discharged into the vascular lumen. Upon arrival, a reduction in intracellular pH and reversing negative membrane potential transpires [7]. Studies indicate an elevation in the synthesis of UA and an increase in the activity of xanthine oxidase during instances of myocardial ischemia [8]. Hyperuricemia frequently co-occurs with metabolic syndrome, comprising hypertension, diabetes, dyslipidemia, and obesity, all of which are established risk factors for coronary heart disease (CHD) [9]. Epidemiological studies have provided evidence that elevated UA levels pose a significant risk factor for the development of cardiovascular disease. Moreover, UA has been associated with various forms of cardiovascular disease-related mortality, including acute, subacute, and chronic presentations. As such, heightened levels of UA may serve as a predictive indicator for acute myocardial infarction (AMI) [10]. The relationship between SUA levels and prognostic outcomes in patients with MI remains a topic of debate within the academic community. The uncertain generalizability of existing studies can be attributed to variations in demographics, temporal factors, and spatial-geographical considerations across different research endeavors. In light of this, the current investigation aimed to address this gap by conducting a comprehensive evaluation and statistical analysis to examine the differences in short- and long-term prognostic consequences between patients with MI who have hyperuricemia and those with normouricemia..

## Methods

In accordance with the PRISMA (Preferred Reporting Items for Systematic Reviews and Meta-analyses) guidelines, a systematic review and meta-analysis were conducted [11]. This study was initially registered on the “PROSPERO” platform (Official Register Number: CRD42023415647).

### Eligibility criteria

We incorporated the PECOs (Population, Exposure, Comparator, Outcome, and Study) criteria to ensure clarity and appropriate study selection. As this study does not involve a randomized controlled trial (RCT), it is crucial to establish well-defined and detailed inclusion criteria to guide the analysis effectively.

The population under investigation encompassed individuals with MI, specifically AMI, ST-segment elevation MI (STEMI), non-ST-segment elevation MI (NSTEMI), silent MI (SMI), and prior chronic ischemic myocardial injury (PCIMI). The included studies examined individuals with varying levels of SUA as the exposure of interest. Comparative analyses were conducted among different SUA levels within the specified population.

The focus of the analysis was on short- and long-term prognostic outcomes associated with MI. To ensure the inclusion of appropriate studies, cohort studies or other similar designs were considered as the preferred study design. By utilizing the PECOs framework, the revised approach provides clear and organized inclusion criteria, addressing any ambiguity regarding eligible study designs.

It is important to note that the inclusion criteria did not impose temporal or spatial constraints, allowing for a comprehensive evaluation of relevant literature without limitation to specific regions or time periods. The findings were based on previously published studies, selected based on predefined ethical considerations to protect participant identity.

This study aims to offer valuable insights to medical professionals, helping them improve patient outcomes and enhance overall quality of life. To maintain methodological rigor, systematic reviews, case reports, editorials, and policy documents were excluded from the analysis. Furthermore, only English-language papers were considered for inclusion in this review, ensuring consistency and facilitating a thorough examination of the selected literature.

### Information sources

To conduct a comprehensive search, we examined electronic bibliographic databases including PubMed, Embase, Web of Science, Medline, Cochrane Library, Emcrae, and Scopus. Additionally, we reviewed the reference sections of relevant publications to identify any supplementary studies that were not initially captured through the aforementioned search approach.

### Search strategy

On 1^st^ Mar. 2023, we searched for medical literature using MeSH in PubMed and Emtree in Embase, and free-text phrases. We adapted headings and wildcards to meet search requirements of different databases. On 1^st^ Apr. 2023, we launched another search for new literature. And we used the “Covidence” for reference management, removing duplicates automatically and manually. The detailed search strategy is in Supplementary Information 1.

### Study selection

Author Jiacheng-Rong and Lei-Huang screened the article titles and abstracts and submitted any content that could not be determined to author Cheng-Fang and Xudong-Chen for joint discussion. For full-text screening, author Lei-Huang recorded any article with uncertain eligibility; the ultimate inclusion was decided jointly with authors Jiacheng-Rong and Cheng-Fang.

### Data extraction

Data from all included studies were extracted into standardized tables in Microsoft Excel Mac Edition (Microsoft Corp., Redmond, WA) (Supplementary Information 2). The following evaluation indicators were extracted from the included studies: 1. publication data: (i) first author's surname and (ii) year of publication; 2. primary statistical data: (i) study site, (ii) study design, (iii) sample size, (iv) patient type, (v) year of follow-up; 3. epidemiological data: a (i) number of exposed versus unexposed, (i i) statistical parameters of outcome: OR, HR, RR, P value, 95% confidence interval (CI). The original authors were contacted for additional data for articles without precise results or with some missing results. Data extraction tables and their contents were confirmed by three authors (Jiacheng-Rong, Lei-Huang and Cheng-Fang).

#### Definition of the various outcome indicators

1. Short-term outcome indicators:

Outcome indicators that occur within 30 days of diagnosis of MI and admission to treatment, including:

i. death within 30 days, including in-hospital and cardiac deaths.
ii. MACE within 30 days, including non-fatal stroke, non-fatal myocardial infarction (NFMI), and cardiovascular death (CD) [12].
iii. Other adverse indicators occurring within 30 days, including atrial fibrillation (AF), acute kidney injury (AKI), myocardial infarction recurrence (RMI), complete atrioventricular (AV) block, coronary artery disease (CAD), coronary prevalence (CP), cardiogenic shock (CS), heart failure (HF), impaired renal function (IRF), major bleeding, renal failure (RF), target vessel revascularization (TVR), ventricular arrhythmia (VR) and Angina pectoris (AP).
iv. Long-term outcome indicators:

Indicators of adverse outcomes occurring more than 30 days after diagnosis of MI and admission to follow-up, including.

i. Death after more than 30 days of follow-up: including all-cause death, cardiac death.
ii. MACE after more than 30 days of follow-up.
iii. other adverse indicators after 30 days of follow-up, AF, AKI, RMI, complete AV block, CAD, CP (refers to the occurrence or prevalence of CAD within a population), CS, HF, IRF, major bleeding, RF, TVR, VR and AP.

#### Definition of high SUA

Hyperuricemia was defined as a SUA level equal to or greater than 420 μmol/L (7.0 mg/dL) in males and equal to or greater than 357 μmol/L (6.0 mg/dL) in females [13].

#### Data Extraction for cohort studies

This review scrutinized the availability of independent data for various cohort time points in the studies under consideration. Following the exclusion of data from duplicate reports, wherein reports of the same cohort in multiple studies were eliminated, the distinct cohort time points were regarded as independent studies in this review.

### Study quality assessment

In this study, the quality of literature for the included studies was assessed using The NOS. As all the literature included in this study consisted of cohort studies, the NOS Cohort Study Scale was employed. The scale encompasses three components: population selection, comparability, and exposure assessment (also known as outcome assessment). Overall, a total of eight entries achieved a score of nine on the scale, indicating high quality based on the assessment criteria [14].

### Synthesis of results

We divide prognostic indicators into short- and long-term indicators based on follow-up duration. To combine data on outcome indicators for hyperuricaemia and normouricaemia, we use a random effects model and RR as the epidemiological indicator. When the articles provide direct epidemiological effect measures, we use a logarithmic approach. To address the varying levels of evidence, HR, RR, and OR are analyzed separately in this study. This approach is adopted considering their distinct interpretations and applications. In instances where the indicators have low prevalence, treatment effects are downscaled, and RR is equated to OR to ensure comparability.

The extracted HR, RR, and OR values were subjected to a multifactorial treatment of confounders. This approach involves considering and adjusting for various factors that may influence the association between the exposure and outcome variables. By accounting for these confounders, the analysis aims to control for their potential impact on the observed associations, thus enhancing the validity and reliability of the results.

In order to address the original hypothesis, all *P*-values pertaining to the same hypothesis were extracted and subjected to a meta-analysis.. The binomial data model, which falls under the generalized linear mixed model, necessitates a substantial amount of computations due to the high-dimensional integration of random effects [15–17], so DerSimonian and Laird random effects models [18] were utilized in a meta-analysis was conducted to determine the combined prevalence and corresponding 95%CI. The statistical heterogeneity between trials was assessed through employment of the X2 test, while the variance resulting from statistical heterogeneity was determined using the I2 statistic. The I2 values corresponding to 25%, 50%, and 75% were categorized as low, medium, and high estimates, respectively. The statistical analysis was conducted utilizing the Stata Statistical Software: Release 17 MP version (StataCorp LP, College Station, TX, USA).

### Assessing publication or reporting bias

We employed funnel plots and conducted Begg's and Egger's tests to evaluate publication bias. However, it is crucial to consider that Cochrane guidelines caution against using funnel plots when the number of included studies is less than 10. In this analysis, the limited number of studies poses a challenge in accurately determining the true effect beyond random chance.

To address the potential publication bias, the research implemented trimming and padding methodologies. These approaches were applied specifically in cases where the results of Begg's or Egger's tests indicated significance with a p-value less than 0.05. By utilizing these techniques, the study aimed to mitigate the impact of publication bias on the overall findings and enhance the reliability of the results.

## Results

### Study selection

**Fig. 1:**
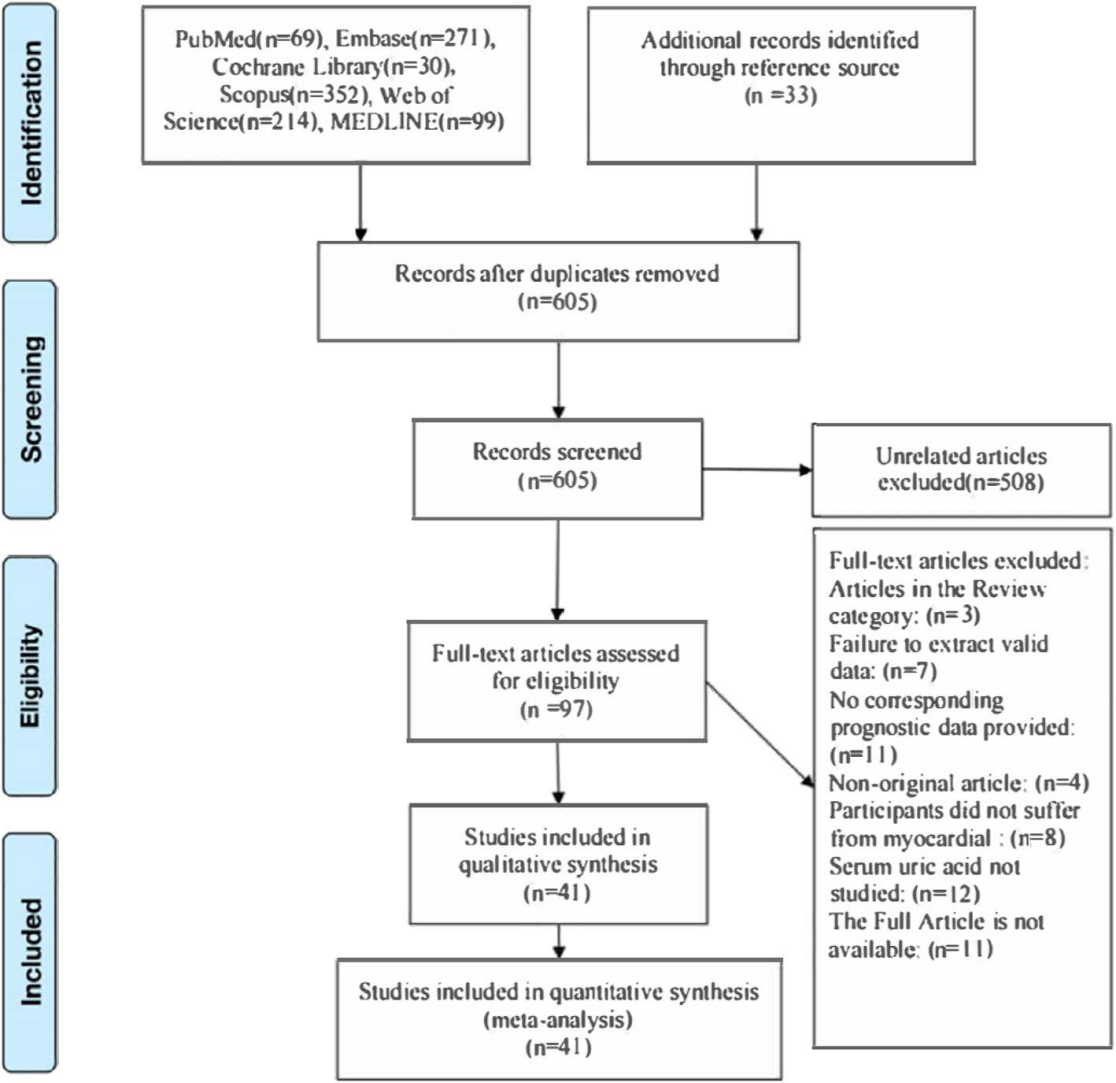
A flow chart depicting the process of study selection along with the rationales for exclusion

The initial database search yielded a total of 1002 articles, along with an additional 33 cited literature sources. After the initial review, 605 articles were identified as potentially relevant and underwent further evaluation. Out of these, 97 articles were deemed eligible for full-text analysis. Following a comprehensive screening process, 55 articles were excluded based on predefined criteria. Ultimately, a total of 35 papers (referenced as papers [19–53]) containing 41 cohorts were included in the study (see Fig. 1). For a detailed list of the excluded studies and the rationale behind their exclusion, please refer to Supplementary Information 3. The study encompassed a total of 41 analyses conducted on a collective sample size of 225,600 patients who had experienced MI. These analyses included two cohort studies, which further contributed to the comprehensive examination of the research question at hand. The substantial number of patients included in these analyses provides a robust foundation for drawing valuable insights and conclusions related to MI.

**Fig. 2:**
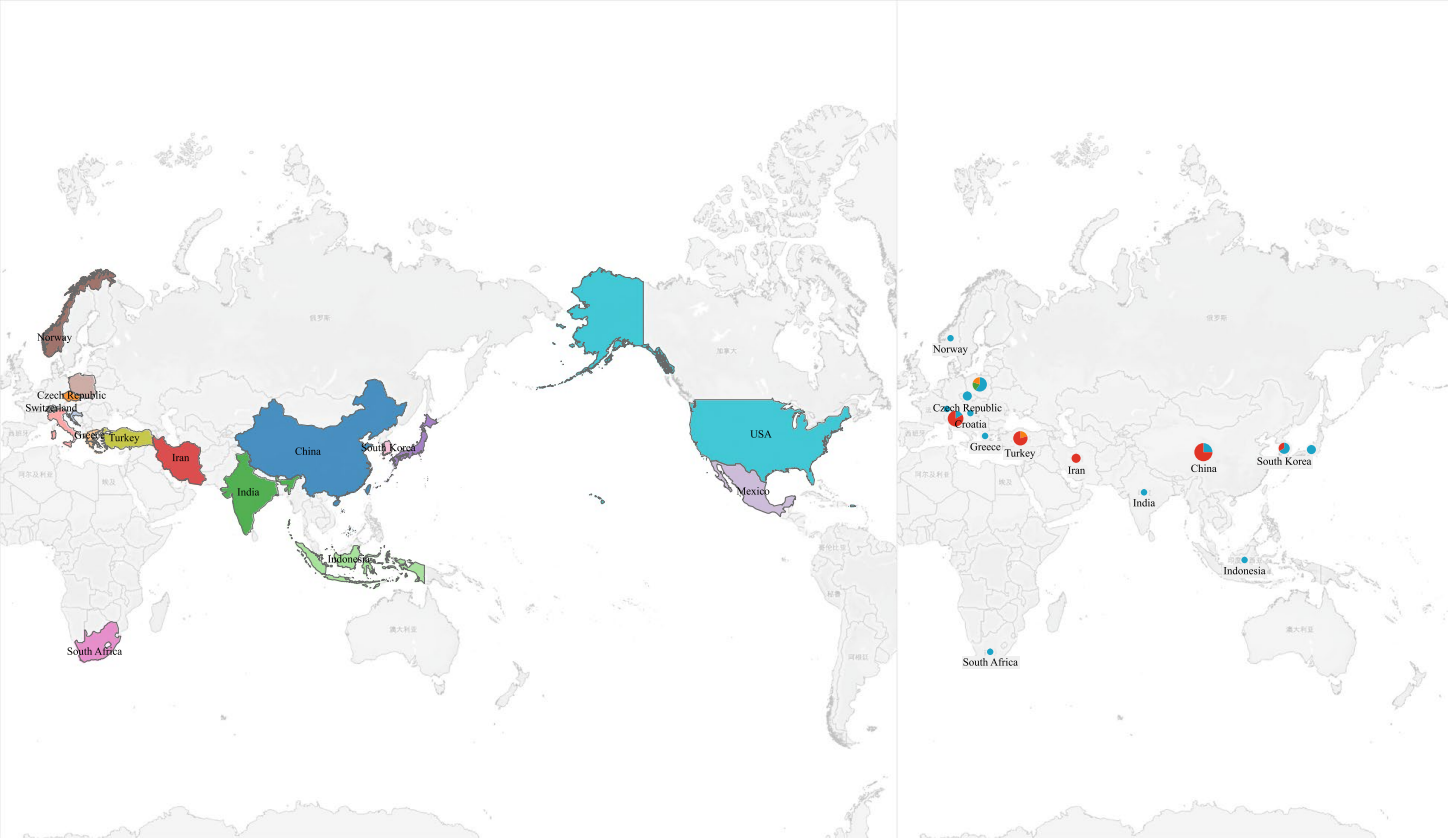
Regional and population characteristics of the included studies

The study encompasses a diverse range of geographic locations, with contributions from 18 distinct countries and regions, such as China, Croatia, the Czech Republic, Greece, India, Indonesia, Italy, Japan, Mexico, Norway, Poland, South Africa, and South Korea. The countries under consideration are Switzerland, Taiwan (region), Turkey, and the United States of America. All individuals who took part in the research were diagnosed patients with MI. The subjects were categorized into four distinct groups based on the type of MI they experienced, namely AMI, STEMI, NSTEMI, and SMI (Fig. 2).

### NOS study quality assessment results

**Fig. 3:**
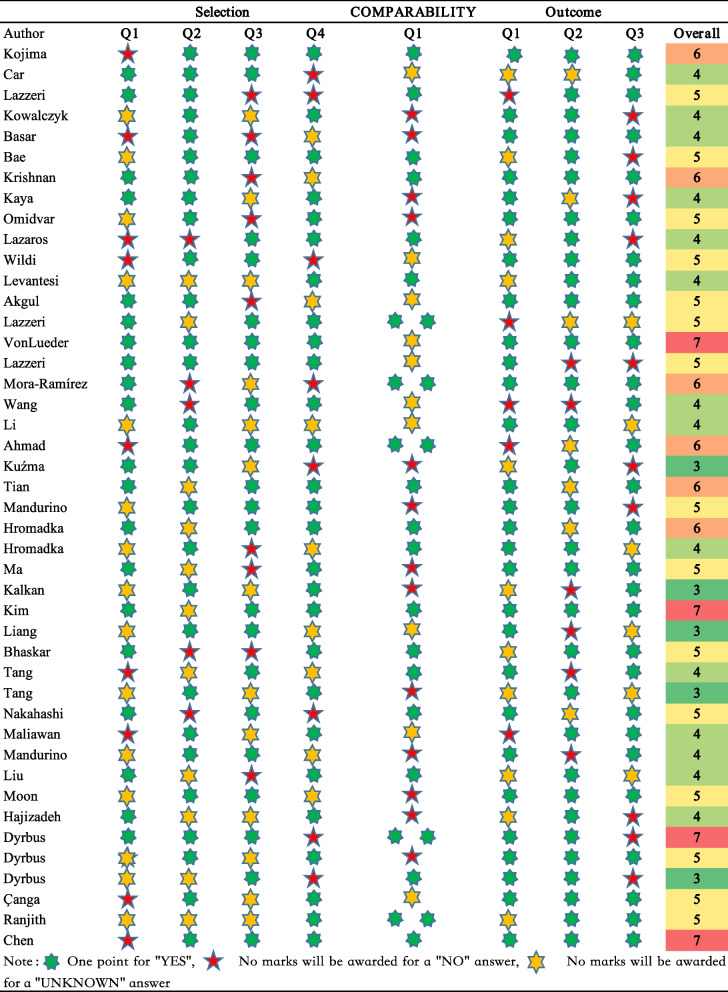
NOS study quality assessment and overall score heat plot

A total of 25 articles had an overall NOS score of 5 or more, which is of medium and above literature quality. An additional 16 articles were below the quality that falls under the average quality score. Overall, the quality of the included literature is in ideal condition (Fig. 3).

### Basic information about the included literature

Two hundred twenty-five thousand six hundred MI patients from 18 different nations and regions were enrolled in 41 studies that made up this review (Supplementary Information 4). The prognostic markers for heart attacks were divided into short-term prognosis and long-term prognosis in this study. There was a total of 19 studies that offered short-term (follow-up 30 days or follow-up outcome occurred in the hospital) outcome indicators, and 37 research that provided long-term (follow-up > 30 days or follow-up outcome happened outside the hospital) outcome data. The outcome signs that occurred most frequently were ACM, MACE, Death, Stroke, and HF.

### Meta-analysis of short-term adverse events

#### The present study aims to investigate the mortality rate in patients diagnosed with MI and hyperuricemia

**Fig. 4:**
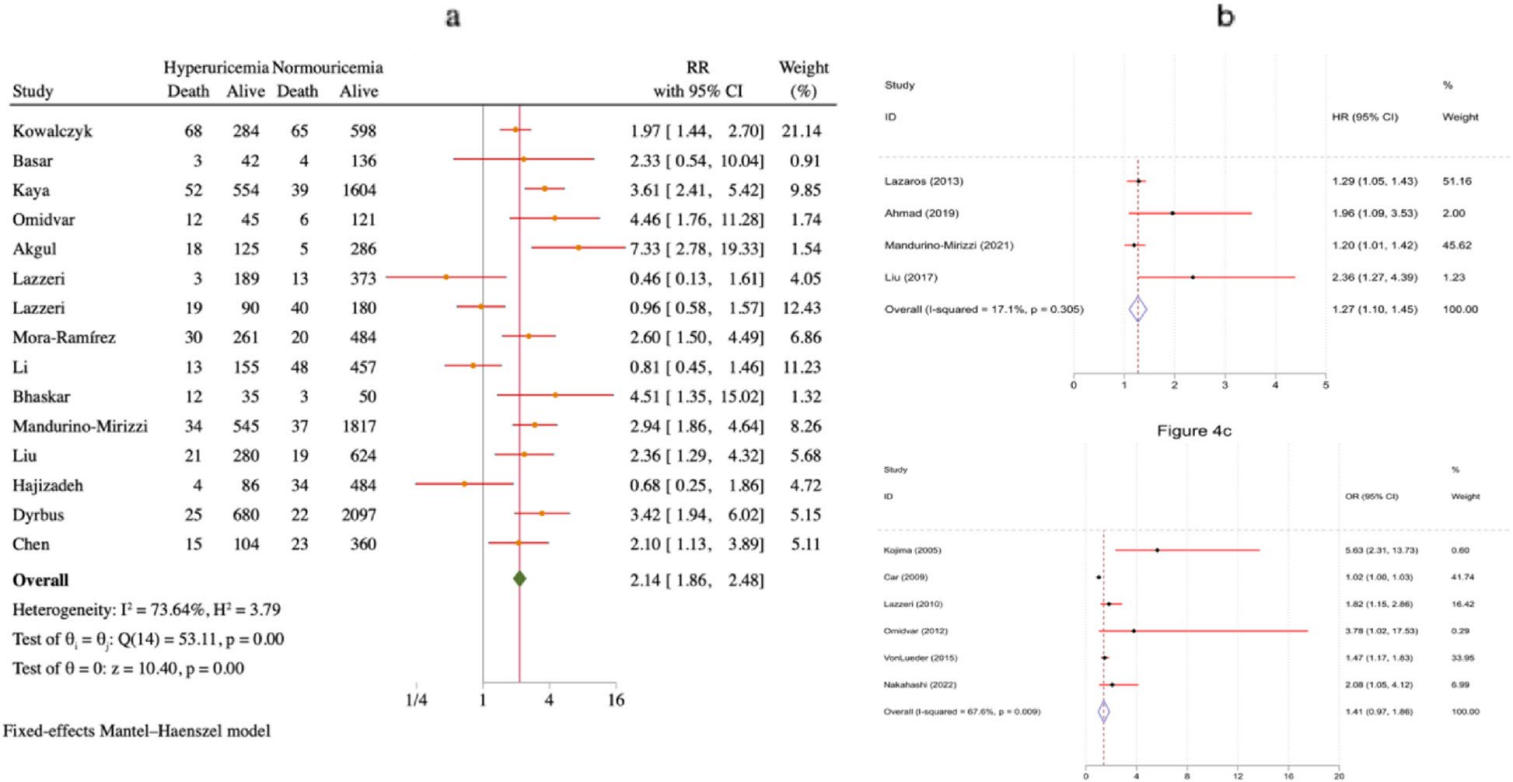
Forest plot of survival in patients with MI with hyperuricemia and normocytic acid blood levels

A meta-analysis was conducted to compare the 30-day mortality rates between patients with MI and high blood UA levels versus those with normal levels. The study yielded noteworthy results, indicating that patients diagnosed with both MI and hyperuricemia had a mortality rate 2.14 times higher (RR = 2.14, 95% CI = 1.86–2.48) than individuals with normouricemia who also experienced MI. This finding is visually represented in Fig. 4a. Separate meta-analytic procedures were employed to analyze HR and RR reported by multiple studies. The research outcomes revealed that individuals with MI and hyperuricemia had a higher frequency of fatal events during the post-treatment phase compared to those with normouricemia and MI. Specifically, the HR for this group was found to be 1.27 (95% CI = 1.01–1.45), as depicted in Fig. 4b. Moreover, the study identified an OR of 1.41 (95% CI = 0.97–1.86) for mortality among patients diagnosed with both MI and hyperuricemia, in comparison to those with normal UA levels and MI, illustrated in Fig. 4c.

#### The incidence of MACE in individuals diagnosed with hyperuricemia and MI has been investigated

**Fig. 5:**
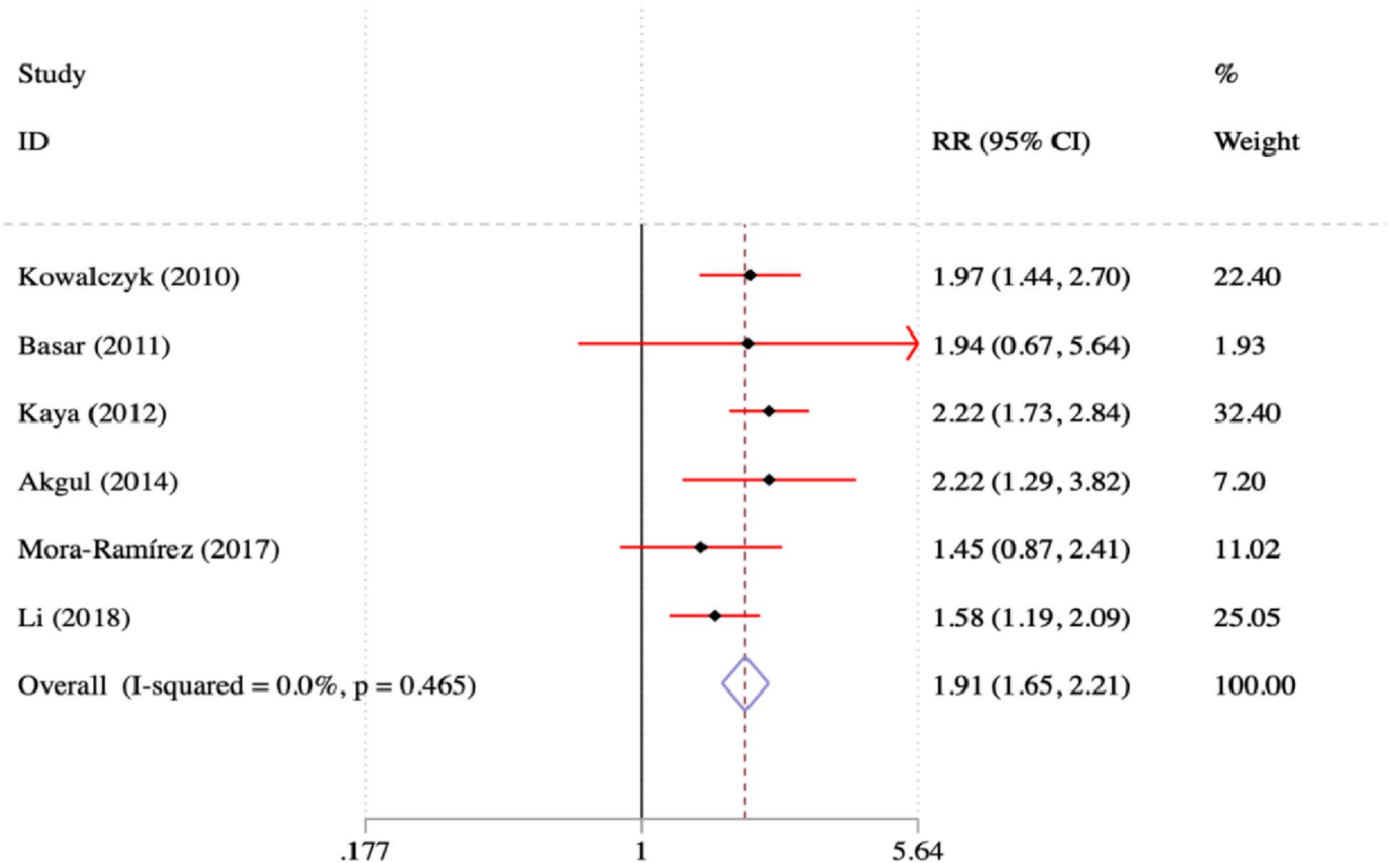
Forest plot of MACE occurrence in patients with MI with hyperuricemia and normocytic acid blood levels

In this study, a meta-analysis was conducted to compare the occurrence of MACE in patients diagnosed with hyperuricemia and MI, as opposed to those with normal UA levels and MI. The results revealed that patients diagnosed with both MI and hyperuricaemia had a 1.91-fold increased risk of experiencing MACE, in comparison to individuals diagnosed with MI and normouricemia. The calculated relative risk (RR) was 1.91, accompanied by a 95% CI ranging from 1.62 to 2.11. These findings are visually represented in Fig. 5.

#### Meta-analysis of patients with MI presenting with other cardiovascular adverse outcomes at different blood UA levels

**Fig. 6:**
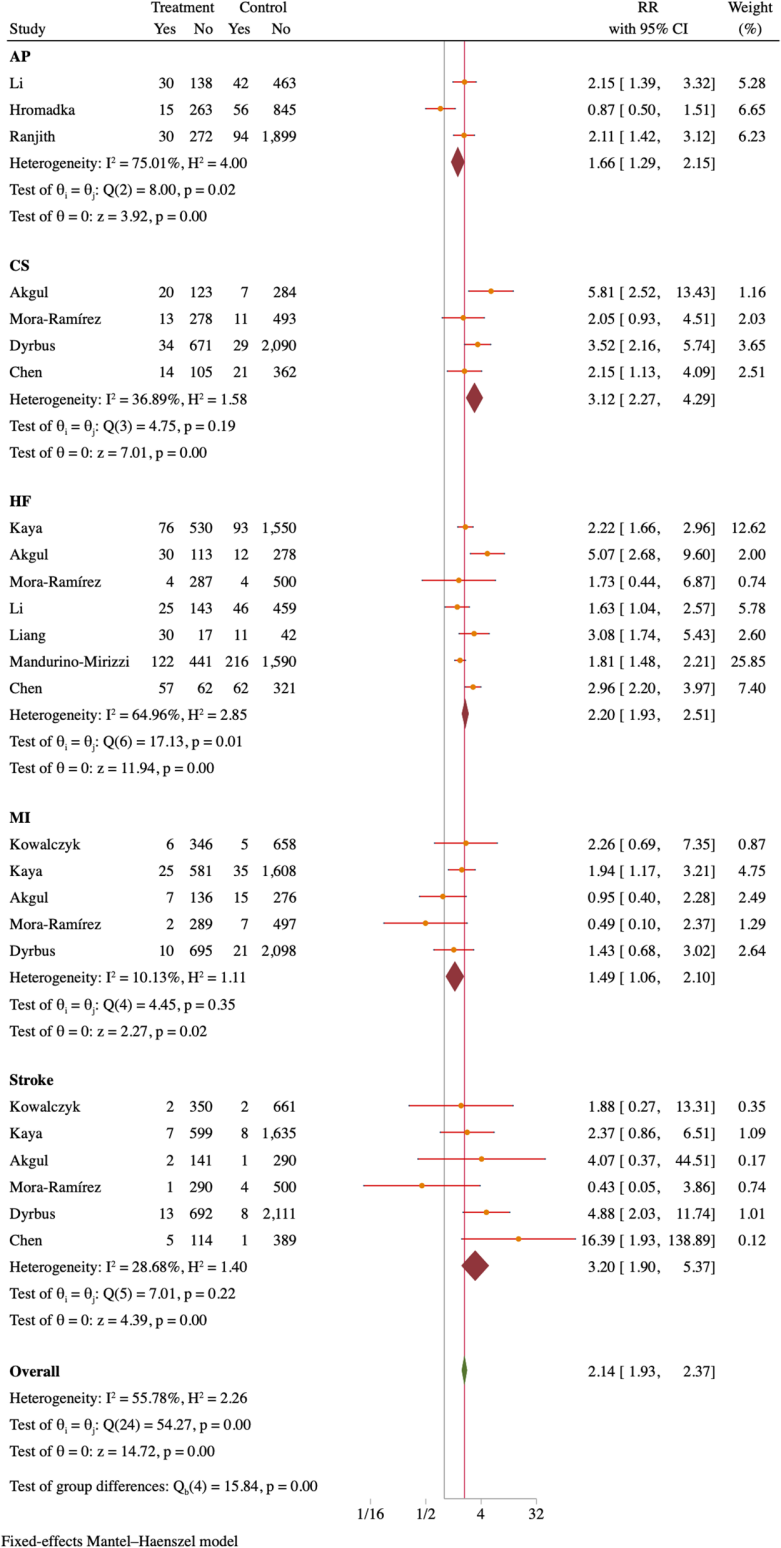
Forest plot of other short-term cardiovascular adverse outcomes

In order to examine the frequency of short-term cardiovascular adverse events, a meta-analysis was conducted. The findings indicate that patients diagnosed with both MI and hyperuricemia are at a heightened risk of experiencing adverse events such as unstable AP compared to those with normal UA levels. The RR was determined to be 1.66, with a 95% CI of 1.29–2.15. Additionally, individuals with MI and hyperuricemia have a significantly higher susceptibility to developing cardiac surgery (CS) in comparison to those with normal UA levels (RR = 2.27, 95% CI = 2.27–4.29). The study also found that patients diagnosed with both hyperuricemia and MI had a significantly increased likelihood of experiencing HF within a 30-day observation period when compared to individuals without hyperuricemia. The relative risk for this group was 2.20, with a 95% CI ranging from 1.93 to 2.51. Furthermore, compared to individuals with normal UA levels during MI, those with hyperuricemia were more prone to experiencing recurrent MI (RR = 1.49, 95% CI = 1.06–2.10). Moreover, the patient cohort under consideration demonstrated a 3.20-fold increase in the likelihood of encountering a stroke (95% CI = 1.90–5.37) compared to patients with normal UA levels during MI (refer to Fig. 6 for detailed illustration).

#### Meta-analysis of patients with MI presenting with non-cardiovascular adverse outcomes at different blood UA levels

**Fig. 7:**
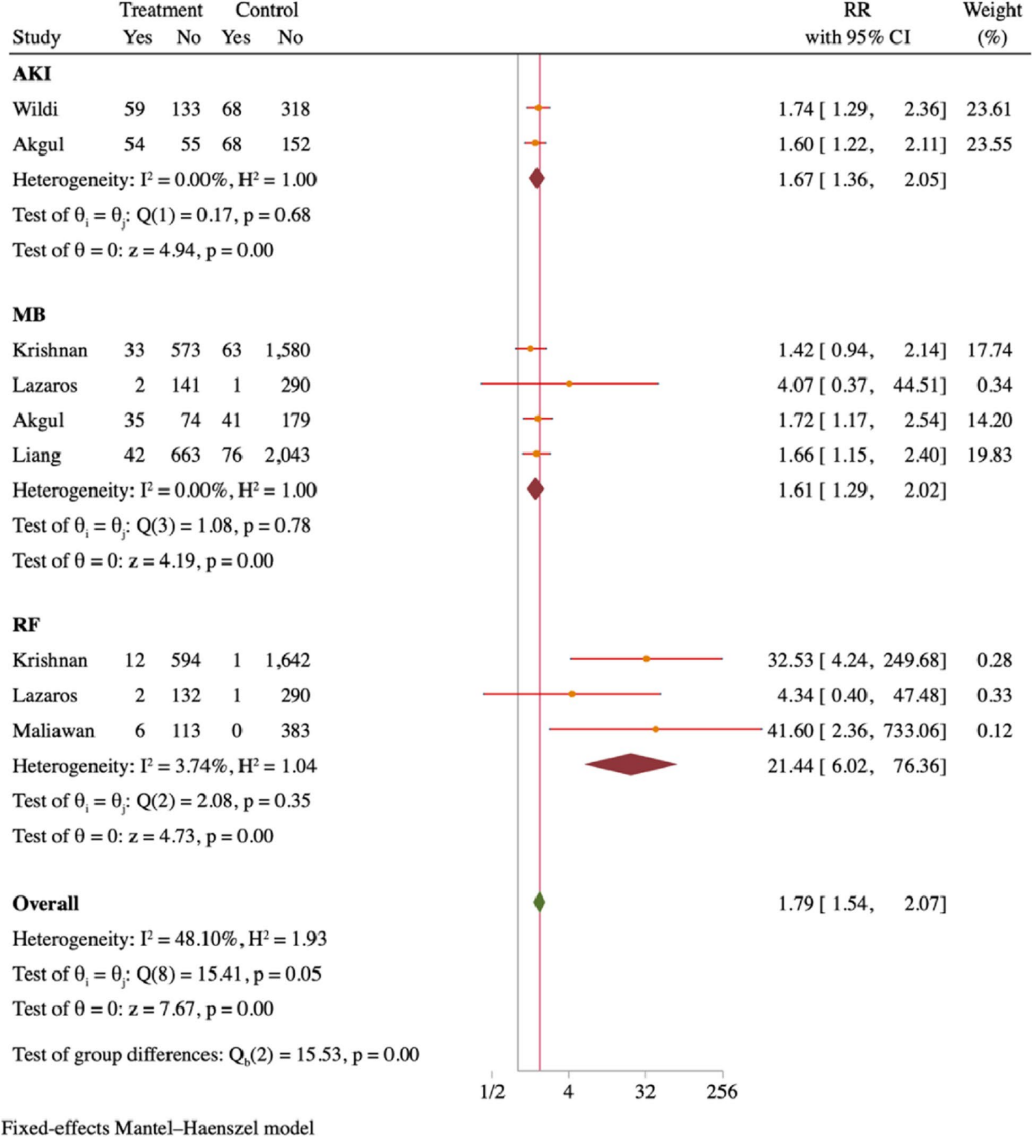
Forest plot of non-cardiovascular adverse outcomes at different blood UA levels

The study findings indicate that individuals diagnosed with MI and hyperuricemia exhibited an increased likelihood of experiencing MB (RR = 1.67, 95% CI. 36–20.5), RF (RR = 1.61, 95% CI = 1.29–2.02), and AKI (RR = 21.44, 95% CI = 6.02–76.36) (detailed in Fig. 7).

### Meta-analysis of Long-term adverse events

#### Association of Hyperuricemia with MI and ACM

**Fig. 8:**
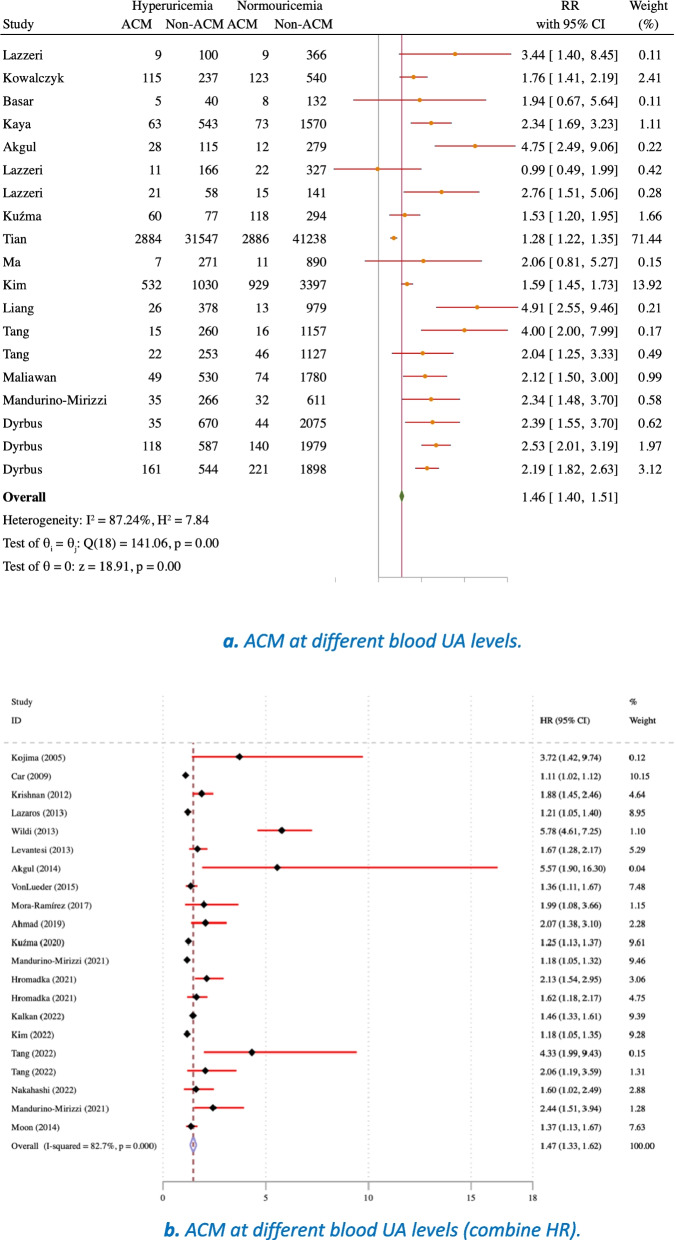
**a** ACM at different blood UA levels. **b** ACM at different blood UA levels (combine HR)

A meta-analysis was conducted to evaluate the association between UA levels and ACM outcomes in patients diagnosed with MI. The study's findings indicate that patients with hyperuricemia have a higher likelihood of experiencing an ACM death compared to those with normal blood UA levels who have suffered from MI (Fig. 8a). A meta-analysis of studies that provided HRs also supports this conclusion, showing that the probability of experiencing an ACM outcome in hyperuricemia with MI is significantly greater than in those with normal UA levels (Fig. 8b).

#### Association of Hyperuricemia with MI and MACE

**Fig. 9:**
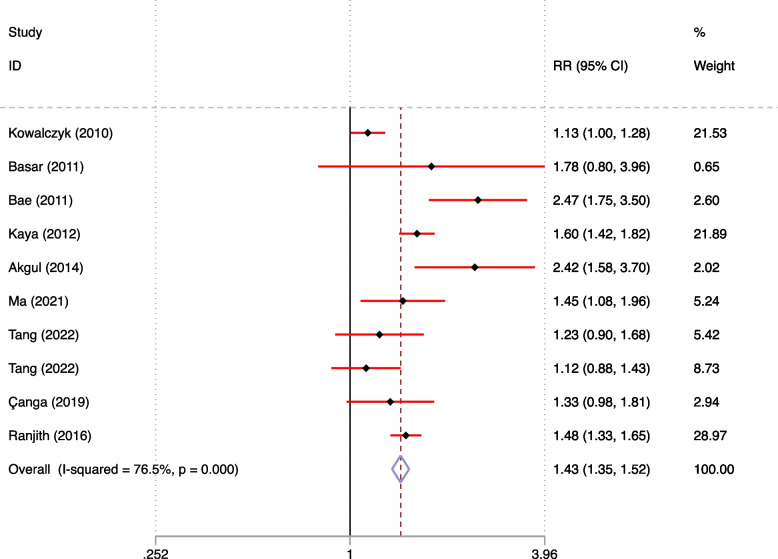
MACE at different blood UA levels

The study findings indicate that the likelihood of MACE in individuals with hyperuricemia and MI was 1.43 times greater than in those with MI and normal blood UA levels (RR = 1.43, 95% CI = 1.35–1.52) (as illustrated in Fig. 9).

#### Meta-analysis of patients with MI presenting with other cardiovascular adverse outcomes at different blood UA levels

**Fig. 10:**
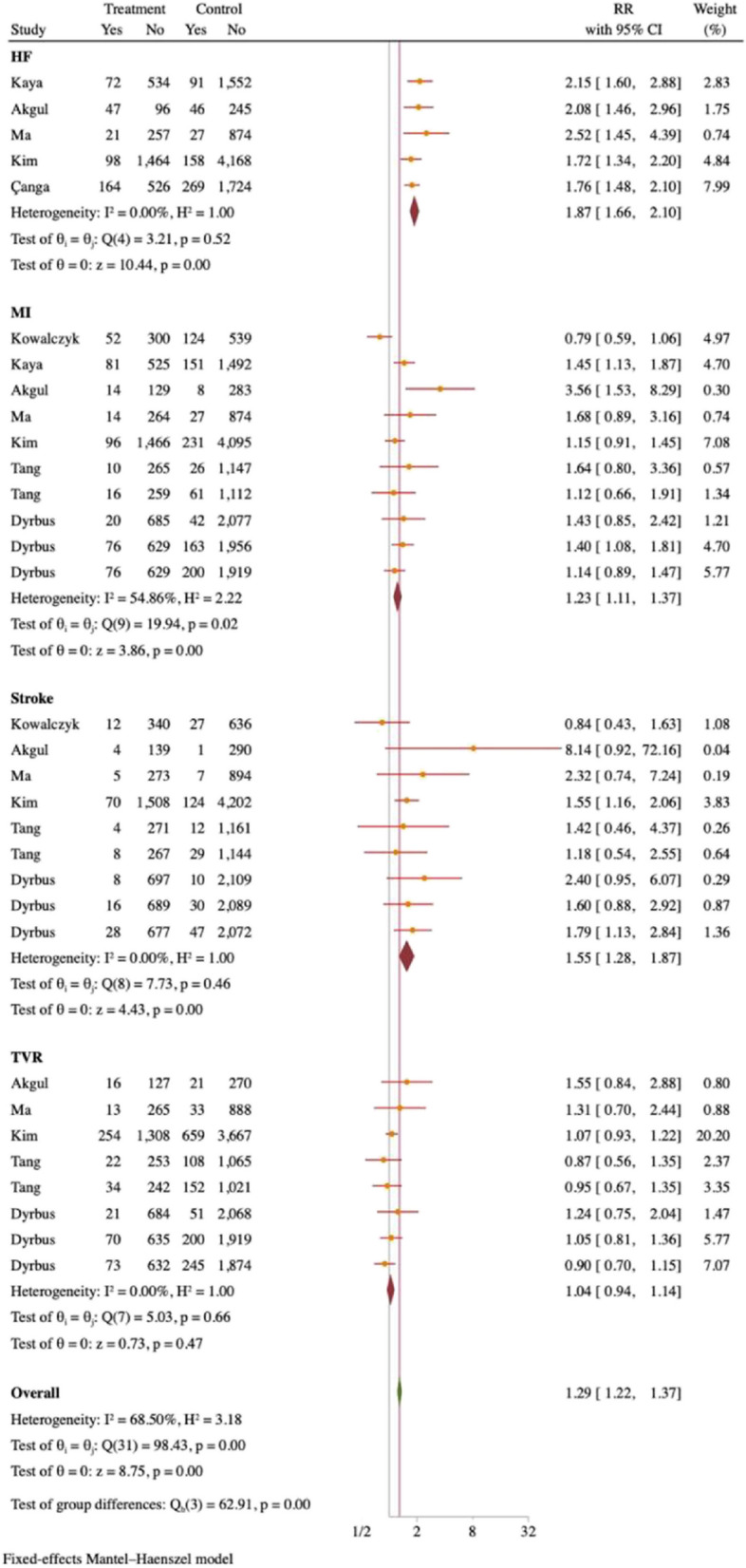
Forest plot of other long-term cardiovascular adverse outcomes at different blood UA levels

A meta-analysis was carried out with the aim of examining further long-term negative cardiovascular event outcomes. The research results suggest that there is a greater propensity for the onset of HF in individuals afflicted with hyperuricemia and MI, as opposed to those with normal levels of UA. The study found a RR of 1.87 and 95% CI of 1.66–2.10 for MI patients with hyperuricemia, showing a 1.23-fold increase in subsequent MI risk compared to those with normal UA levels (RR 1.23, 95% CI 1.11–1.37). These patients also had a 1.55-fold increase in stroke risk (RR 1.55, 95% CI 1.28–1.87). Patients who have been diagnosed with both MI and hyperuricemia are at a slightly higher risk of experiencing a TVR compared to those with normal UA levels. Specifically, there is a 1.04-fold increase in likelihood, with a 95%CI ranging from 0.94 to 1.14. Individuals who have been diagnosed with both MI and hyperuricemia have a higher probability of experiencing additional unfavorable cardiovascular events, with a 1.29-fold increase in comparison to those who have MI and normal UA levels. This conclusion is based on the statistical analysis of data, with a relative risk of 1.29 and a 95%CI ranging from 1.22 to 1.37, as illustrated in Fig. 10.

### Sensitivity and publication bias analysis

After sensitivity analysis using an exclusion-by-exclusion approach, we found no significant change in the long-term and short-term adverse outcomes. We also excluded articles with NOS quality scores below 5; no significant differences were observed. These minor changes suggest that the meta-analysis is robust and reliable. We also performed tests for publication bias with all studies having a p-value of > 0.05 for Egger test. For some studies with results close to 0.05, we found only weak changes in the results after using trimming and padding methods, indicating that the meta-analysis was robust (Supplementary information 5).

## Discussion

The existing epidemiological research indicates that elevated levels of SUA pose a noteworthy risk for cardiovascular disease, wherein oxidative stress assumes a quite critical pathophysiological function. In addition, research has also demonstrated that the utilization of a xanthine oxidoreductase inhibitor can effectively reduce SUA concentrations and provide safeguarding advantages in scenarios characterized by oxidative stress, such as instances of ischemia–reperfusion injury and cardiovascular disease [54–56]. The detection of UA may precede that of other cardiac markers, including cardiac troponins. Spectral unipolar atrial electrograms (SUA) have been identified as a viable early indicator of myocardial ischemia [57, 58]. As such, it has been deemed an efficacious approach for forecasting the concomitant occurrence of MI and troponins.

Prior research has investigated the prognostic properties of relevant biomarkers associated with atherosclerotic cardiovascular disease [59–61]. The clinical utility of SUA in patient assessment is apparent, however, the impact of SUA on drug therapy and prognosis enhancement is yet to be fully elucidated [57]. Following a comprehensive examination and statistical analysis of 41 studies, our findings indicate that hyperuricemia is associated with a higher likelihood of short-term adverse outcomes compared to long-term adverse outcomes in individuals diagnosed with MI. The study found a significant increase in risk of short-term (2.14x) and long-term all-cause mortality (1.47x) in MI patients with elevated SUA levels. These patients also exhibited a higher frequency of MACE over both short and long-term periods. Hyperuricemia impacts cardiovascular prognosis and risks. The meta-analysis RR's 95%CI do not encompass non-significant values. The elevated SUA levels have an impact on future mortality rates, MACE occurrence, and other unfavorable cardiovascular outcomes in MI patients.

The meta-analysis exhibits some constraints. Initially, it should be noted the absence of a specialized randomized controlled trial for this investigation necessitated the utilization of solely observational studies for data extraction, which may potentially introduce a risk of associated bias. Despite the extraction of adjusted epidemiological effect values for RR and HR and the subsequent meta-analysis of these values, it is crucial to recognise that a limited quantity of residual confounding factors may exist. Furthermore, it is improbable that the methodology employed in every study was entirely uniform. The studies exhibited variability with regards to factors such as age range, gender distribution, and medical history of the participants. Thus, it should be noted that this meta-analysis has an inherent limitation due to the possibility of heterogeneity among the included studies. And the HSUA cut-off values exhibited variability across the studies, ranging from 5.4 mg/dL to 7.5 mg/dL. However, the majority of the studies (10 out of 13) employed HSUA cut-off levels within the range of 6 mg/dL to 7 mg/dL. All possible endeavours were undertaken to evaluate the cumulative impact by excluding research studies where the HSUA threshold varied significantly from that of other studies.

The present investigation exhibits certain notable strengths. The meta-analysis incorporated studies with considerable sample sizes. A systematic evaluation is required to appraise the quality of individual studies. Nonetheless, there exists a lack of consensus regarding the most suitable approach for evaluating the quality of a study. The decision was made to evaluate the aforementioned studies utilising the Newcastle–Ottawa Scale (NOS), a tool that encompasses three crucial components for the evaluation of cohort studies. The adverse events were classified into short-term and long-term categories, and an effort was made to encompass nearly all conceivable adverse events, rendering this investigation a preeminent and all-encompassing reference.

The study has several notable strengths. Firstly, a comprehensive and extensive search strategy was employed, encompassing both primary articles and relevant cited literature, thus ensuring a thorough exploration of the topic. Additionally, the inclusion of a large sample size comprising 225,600 patients enhances the statistical power and generalizability of the findings. The incorporation of cohort studies further contributes valuable longitudinal data, providing insights into the long-term effects and outcomes related to MI.

However, there are also certain limitations to consider. Despite efforts to address publication bias through methods such as funnel plots and statistical tests, the limited number of included studies may still introduce some uncertainty regarding the true effect beyond random chance. Heterogeneity among the included studies is another potential limitation that could impact the overall results. Conducting subgroup analyses or sensitivity analyses could have helped explore this further. Additionally, variations in data quality across different studies could influence the reliability and consistency of the results.

Comparing our study to other similar meta-analyses, we stand out due to our significantly large sample size and the inclusion of cohort studies. These factors enhance the robustness and reliability of our assessment of MI. However, it is important to acknowledge that other meta-analyses may have addressed certain limitations, such as publication bias, in more comprehensive ways by including a greater number of studies. Moreover, differences in the research question, inclusion criteria, and methodology across studies can lead to variations in results and conclusions. Therefore, a collective consideration of the findings from multiple meta-analyses is crucial for a comprehensive understanding of the topic.

## Conclusion

The study provides evidence that patients with MI and elevated SUA levels have higher rates of short and long-term mortality, MACE, and other unfavorable outcomes. These findings suggest that SUA may play a role in the progression of atherosclerosis and could serve as a novel prognostic indicator for monitoring individuals following AMI. Incorporating SUA as a prognostic metric in clinical practice has the potential to significantly improve risk stratification and enhance the precision of existing techniques. This approach could lead to more effective medical interventions, reduced healthcare costs, and improved patient well-being by lowering readmission rates and medical expenses. It is important to note that ethical approval or patient consent was not required for this study as it relied on previously published research.

### Supplementary Information

**Additional file 1:** **Figure SA.** Funnel plot of publication bias for short-term adverse effects. *Note: *The Egger test *p*-value was 0.053. We adjusted for this using the trim and fill method and the results showed that, after adjustment, the Egger test *p*-value was 0.055.There was only a weak change, suggesting that the results of the meta-analysis on short-term mortality were robust. **Figure SB.** Funnel plot of publication bias for MACE.*Note: *The *p*-value for the Egger's test was 0.079, indicating that our results for the MACE meta-analysis were reliable and robust. **Figure SC.** Funnel plot of publication bias for other cardiovascular adverse outcomes.* Note: *The Egger test *p*-value was 0.0501. We adjusted for this using the trim and fill method and the results showed that, after adjustment, the Egger test *p*-value was 0.0512. There was only a weak change, suggesting that the results of meta-analysis on other cardiovascular adverse outcome were robust. **Figure SD.**Funnel plot of publication bias for long-term adverse effects.* Note:*The *p*-value for the Egger's test was 0.059, indicating that our results for the MACE meta-analysis were reliable and robust. **S1 Table 1.** Mesh Term & Entry Term. **S1 Table 2.** Scopus Serach Strategy. **S1 Table 3.** Cochrane Serach Strategy. **S1 Table 4.** MEDLINE Serach Strategy. **S1 Table 5.** Embase Serach Strategy. **S1 Table 6.** Global Health Serach Strategy. **S2 Table 1.** Data extraction form. **S2 Table 2.** Abbreviation. **S3 Table 1.** Article exlude reason. **S4 Table 1.** Characteristics of all literature included in this study and the outcome variables included.

## Data Availability

All data generated or analyzed during this study are extracted from published papers. And all data generated or analyzed during this study are included in this published article [and its supplementary information files].
