# Correction: Association of serum uric acid with prognosis in patients with myocardial infarction: an update systematic review and meta-analysis

**DOI:** 10.1186/s12872-023-03637-6

**Published:** 2023-12-11

**Authors:** Jiacheng Rong, Cheng Fang, Xudong Chen, Chaokun Hong, Lei Huang

**Affiliations:** 1 Cardiovascular Department, Ningbo Hangzhou Bay Hospital, Qianwan New District, Ningbo, Zhejiang China; 2 Department of Urologic Surgery, Ningbo Urology and Nephrology Hospital, Ningbo Yinzhou No.2 Hospital, Ningbo, Zhejiang China; 3 MRC Centre for Global Infectious Disease Analysis Faculty of Medicine, Imperial College, London, UK

**Correction to: BMC Cardiovascular Disorders (2023) 23:1**

10.1186/s12872-023-03523-1

Following publication of the original article [1], the order of the author names has been updated.

The original article has been corrected.

