# Supplementary material for: Association of serum uric acid with prognosis in patients with myocardial infarction: an update systematic review and meta-analysis
Source: BMC Cardiovasc Disord. 2023 Oct 17;23:512. doi: 10.1186/s12872-023-03523-1 (PMC10583382; doi:10.1186/s12872-023-03523-1)
Supplement: Supplementary file 1 — Additional file 1: Figure SA. Funnel plot of publication bias for short-term adverse effects. Note: The Egger test p-value was 0.053. We adjusted for this using the trim and fill method and the results showed that, after adjustment, the Egger test p-value was 0.055.There was only a weak change, suggesting that the results of the meta-analysis on short-term mortality were robust. Figure SB. Funnel plot of publication bias for MACE.Note: The p-value for the Egger's test was 0.079, indicating that our results for the MACE meta-analysis were reliable and robust. Figure SC. Funnel plot of publication bias for other cardiovascular adverse outcomes. Note: The Egger test p-value was 0.0501. We adjusted for this using the trim and fill method and the results showed that, after adjustment, the Egger test p-value was 0.0512. There was only a weak change, suggesting that the results of meta-analysis on other cardiovascular adverse outcome were robust. Figure SD.Funnel plot of publication bias for long-term adverse effects. Note:The p-value for the Egger's test was 0.059, indicating that our results for the MACE meta-analysis were reliable and robust. S1 Table 1. Mesh Term & Entry Term. S1 Table 2. Scopus Serach Strategy. S1 Table 3. Cochrane Serach Strategy. S1 Table 4. MEDLINE Serach Strategy. S1 Table 5. Embase Serach Strategy. S1 Table 6. Global Health Serach Strategy. S2 Table 1. Data extraction form. S2 Table 2. Abbreviation. S3 Table 1. Article exlude reason. S4 Table 1. Characteristics of all literature included in this study and the outcome variables included. [file 12872_2023_3523_MOESM1_ESM.zip › Supplementary Information 4.doc]

***S4 Table 1.*** *Characteristics of all literature included in this study and the outcome variables included.*

| **Author** | **Year** | **FY** | **Location** | **Participant** | **N** | **Short-term outcome variables** | **Long-term outcome variables** | **Reference** |
| --- | --- | --- | --- | --- | --- | --- | --- | --- |
| Kojima | 2005 | 2002 | Japan | AMI | 1124 | Death; MACE; | ACM; | 19 |
| Car | 2009 | 1996-2001 | Croatia | AMI | 621 |  | ACM; | 20 |
| Lazzeri | 2010 | 2008 | Italy | STEMI | 466 |  | ACM; | 21 |
| Kowalczyk | 2010 | 2000-2007 | Poland | AMI+IRF | 1015 | MACE; Stroke; MI; | MACE; Stroke; MI; Death; | 22 |
| Basar | 2011 | 2010-2011 | Turkey | STEMI | 190 | MACE; | MACE; Death; | 23 |
| Bae | 2011 | 2005-2008 | South Korea | AMI | 850 |  | MACE; | 24 |
| Krishnan | 2012 | 1974-1979 | USA | AMI | 4352 |  | ACM; Stroke; CHDM; CI | 25 |
| Kaya | 2012 | 2003-2009 | Turkey | STEMI | 2568 | MACE; Stroke; TVR; MI; VA; HF; RF; CABRTP; MB; AF | ACM; MACE; MI; HF; | 26 |
| Omidvar | 2012 | - | Iran | STEMI | 184 |  |  | 27 |
| Lazaros | 2013 | 2009-2011 | Greece | AMI | 375 |  | ACM; | 28 |
| Wildi | 2013 | 2006-2009 | Switzerland | AMI | 829 |  | ACM; | 29 |
| Levantesi | 2013 | 2001 | Italy | AMI | 10840 |  | ACM; MACE; | 30 |
| Akgul | 2014 | 2010-2012 | Turkey | STEMI | 464 | MACE; Stroke; TVR; MI; CS; HF; RF; MB; AF; | ACM; MACE; Stroke; MI; Death; HF; TVR; | 31 |
| Lazzeri | 2015 | 2006-2013 | Italy | STEMI | 1505 | AKI; | Death; | 32 |
| VonLueder | 2015 | - | Norway | AMI | 28771 | Death; | ACM; HF; | 33 |
| Lazzeri | 2015 | 2006-2013 | Italy | STEMI | 1505 | MACE; MB; AKI; | ACM; | 34 |
| Mora-Ramírez | 2017 | 2006-2012 | Mexico | STEMI | 795 | MACE; Stroke; MI; CS; HF; | ACM; | 35 |
| Wang | 2017 | 2010-2013 | China | STEMI | 701 | MACE; |  | 36 |
| Li | 2018 | 2013-2015 | China | AMI | 673 | HF; AP; |  | 37 |
| Ahmad | 2019 | 1988-1994 | USA | SMI | 6323 | Death; | ACM; | 38 |
| Kuźma | 2020 | 2017-2018 | Poland | NSTEMI | 549 |  | ACM; | 39 |
| Tian | 2021 | 2006-2012 | China | AMI | 85503 |  | ACM; | 40 |
| Mandurino | 2021 | 2006-2017 | Italy | STEMI | 2369 |  | ACM; | 41 |
| Hromadka | 2021 | 2006-2018 | Czech Republic | AMI | 5196 |  | ACM; Death; | 42 |
| Hromadka | 2021 | 2006-2018 | Czech Republic | AMI | 5196 | AP; | ACM; Death; | 42 |
| Ma | 2021 | 2015-2019 | China | STEMI | 23460 |  | ACM; MACE; Stroke; MI; HF; TVR; | 43 |
| Kalkan | 2022 | 2015-2020 | Turkey | STEMI | 5112 |  | ACM; | 44 |
| Kim | 2022 | 2004-2009 | South Korea | AMI | 10719 |  | ACM; Stroke; MI; Death; HF; TVR; | 45 |
| Liang | 2023 | 2014-2017 | China | STEMI | 1653 |  | ACM; | 46 |
| Bhaskar | 2016 | 2013-2014 | India | AMI | 100 | VA; HF; |  | 47 |
| Tang | 2022 | 2013-2015 | China | STEMI | 1448 |  | ACM; MACE; Stroke; MI; TVR; | 48 |
| Tang | 2022 | 2013-2018 | China | STEMI | 1448 |  | ACM; MACE; Stroke; MI; Death; TVR; | 49 |
| Nakahashi | 2022 | 2013-2014 | Japan | AMI | 1695 |  | ACM; Death; | 50 |
| Maliawan | 2017 | 2016-2017 | Indonesia | AMI | 87 | MACE; |  | 51 |
| Mandurino | 2021 | 2006-2017 | Italy | STEMI | 2433 | HF; | Death; | 52 |
| Liu | 2017 | 2006-2012 | Taiwan | STEMI | 951 |  | Death; | 53 |
| Moon | 2014 | 2007-2013 | South Korea | STEMI | 496 |  | ACM; | 54 |
| Hajizadeh | 2016 | 2012-2014 | Iran | STEMI | 680 |  |  | 55 |
| Dyrbus | 2021 | 2006-2016 | Poland | AMI | 2824 | Stroke; TVR; MI; CS; MB; | ACM; Stroke; MI; TVR; | 56 |
| Dyrbus | 2021 | 2006-2016 | Poland | AMI | 2824 |  | ACM; Stroke; MI; TVR; | 56 |
| Dyrbus | 2021 | 2006-2016 | Poland | AMI | 2824 |  | ACM; Stroke; MI; TVR; | 56 |
| Çanga | 2019 | 2010-2015 | Turkey | NSTEMI | 697 |  | MACE; HF; | 57 |
| Ranjith | 2016 | 2006-2014 | South Africa | AMI | 2683 | AP; | MACE; | 58 |
| Chen | 2012 | 2005-2010 | China | STEMI | 502 | Stroke; VA; CS; HF; RF; CABRTP; |  | 59 |
| Note: FY: Follow-up year; ACM: All-cause mortality; AF: Atrial fibrillation; AKI: Acute kidney injury; AP: Angina pectoris; CABRTP: Complete atrioventricular block requiring transient pacemaker; CHDM: Coronary heart disease death; CI: Coronary incidence; CS: Cardiogenic shock; HF: Heart Failure; IRF: Impaired renal function; MACE: Major adverse cardiac events; MB: Major bleeding; MI: Myocardial infarction; N: Number of participants; RF: Renal failure; TVR: Target vessel revascularization; VA: Ventricular arrhythmias. | | | | | | | | |
